# The effect of turmeric on lipid profile, malondialdehyde, liver echogenicity and enzymes among patients with nonalcoholic fatty liver disease: a randomized double blind clinical trial

**DOI:** 10.1186/s13098-021-00731-7

**Published:** 2021-10-18

**Authors:** Maryam jarhahzadeh, Pezhman Alavinejad, Farnaz Farsi, Durdana Husain, Afshin Rezazadeh

**Affiliations:** 1 Department of Nutrition, Faculty of Paramedicine, Ahvaz Jundishapur University of Medical Sciences, Ahvaz, Iran; 2 Alimentary Tract Research Center, Ahvaz Jundishapur University of Medical Sciences, Azadegan Avenue, Ahvaz, Iran; 3 Colorectal Research Center, Iran University of Medical Sciences, Tehran, Iran; 4 Department of Nutrition, School of Paramedicine, Ahvaz Jundishapur University of Medical Sciences, Ahvaz, Iran; 5 Faculty of Medicine, Ahvaz Jundishapur University of Medical Sciences, Ahvaz, Iran

**Keywords:** Turmeric, NAFLD, Liver transaminases, Metabolic syndrome, Curcumin

## Abstract

**Background:**

Nonalcoholic fatty liver disease (NAFLD) is one of the most common causes of liver transaminases elevation and a global health concern.

**Purpose:**

This study designed to evaluate the effects of turmeric rhizomes (Curcumalonga Linn.) on liver enzymes, Lipid profiles and Malondialdehyde (MDA) in patients with NAFLD.

**Study design:**

Randomized double-blind placebo controlled trial.

**Methods:**

64 cases of NAFLD randomly assigned to receive either turmeric (2 gr/day) or placebo for 8 weeks. The changes of liver transaminases, lipid profiles and MDA were measured before and after study period and compared between two groups (IRCT 2015092924262N1).

**Results:**

At the end of the study, the Turmeric group showed a significant reduction in liver enzymes (AST before 26.81 ± 10.54 after 21.19 ± 5.67, P = 0.044, ALT before 39.56 ± 22.41, after 30.51 ± 12.61, P = 0.043 and GGT before33.81 ± 17.50, after 25.62 ± 9.88, P = 0.046) compared with the placebo group. The serum levels of triglycerides, LDL, HDL and MDA had also a significant decrease among turmeric group as compared to baseline while there was no significant change in placebo group (P < 0.05). The serum cholesterol, VLDL level and sonographic grades of NAFLD had not any significant change in both groups.

**Conclusion:**

In conclusion this study suggests that daily consumption of turmeric (and its active phenolic ingredients as curcumin) supplementation could be effective in management of NAFLD and decreasing serum level of liver transaminases.

## Introduction

Nonalcoholic fatty liver disease (NAFLD) is a clinico-pathologic condition which characterized with accumulation of lipids in the liver [1, 2]. This condition is one of the most common causes of liver transaminases elevation as a global health concern and unrelated to alcohol consumption [3, 4]. NAFLD contains a wide range of disorders from simple steatosis to nonalcoholic steatohepatitis (NASH), liver cirrhosis and hepatocellular carcinoma [5, 6]. Previously NAFLD has been considered as a benign condition while more recent studies have proven it as a serious and progressive disorder with raising prevalence twice in the past two decades [7, 8].

There is not any accurate estimate of NAFLD prevalence among different communities because this condition could be present in the absence of any transaminase rising even among diabetics and usually is asymptomatic [9, 10]. However, it is reported to involve 57% of obese persons, 70% of diabetics and even 90% of morbid obese population with an increasing course [11]. Fat accumulation in liver has a key role in pathogenesis of other morbid conditions such as heart failure or diabetes mellitus and could be considered as an additional feature of the metabolic syndrome, with specific hepatic insulin resistance [12, 13]. The pathogenesis of NAFLD and NASH appears to be multifactorial and many mechanisms have been proposed as possible causes of fatty liver infiltration [13, 14]. The well knows factors in induction of fatty liver include fatty acids, TNFα and adiponectin and imbalance of their concentration [15]. In this setting, the oxidative stress has a key role in progression of NAFLD [16, 17]. Oxidative stress also triggers production of inflammatory cytokines, causing inflammation and a fibrogenic response [18, 19].

The current therapeutic strategies for NASH treatment are mostly directed toward correction and modifying risk factors such as obesity, diabetes mellitus and hyperlipidemia with especial concern toward oxidative stress [19, 20]. In this regard, several natural herbal products have the potential to be hepatoprotective and therefore can be used to treat acute and chronic liver diseases [21–23]. One of these hepatoprotective herbs is Turmeric (Curcuma longa) or its constituent which has been used not only as a dietary spice but also as a traditional medicine for many centuries and its properties have been reported in the literature [20, 24–26]. Curcumin (dihydroferuloyl-methane), a natural polyphenol, is a biologically active phytochemical substance extracted from turmeric (*Curcuma longa* L.) [27–29]. *Curcuma longa L*. seems to posse a surprisingly wide range of pharmacologic actions, including anti-carcinogenic, anti-inflammatory, antioxidant, and immunomodulatory activities [28, 30]. Potential hepatoprotective activity of Turmeric and its constituent have been known in Indian traditional medicine hundreds years ago. Recent evidences have also demonstrated the efficacy of curcumin in liver function improvement [21, 30, 31]. Moreover, available evidence showed that supplementation with Curcumin may be effective for liver diseases [30–32]. However, some studies did not show positive effects of curcumin on liver function [33]. Due to inconsistent findings, we cannot make a certain decision on the effects of curcumin on liver enzymes. According to recent evidences, potential benefits and availability of turmeric (curcumin), this randomized clinical trial has been designed to evaluate any potential role of oral turmeric on the liver enzymes, lipid profile, malondialdehyde (MDA) and the grade of hepatic steatosis.

## Materials and method

### Study design

This clinical trial was approved by the Ethical Committee of Ahvaz Jundishapur University of Medical Sciences (IR.Ajums.rec.1394.104) and study registered at IRCT.ir (IRCT 2015092924262N1). A written informed consent was signed by all of the participants at the beginning of the study.

In this clinical trial, during 3 months all of the NAFLD patients who attend at the outpatient hepatology clinic of Ahvaz Imam Hospital as a tertiary center included.

Inclusion criteria include clinical diagnosis of NAFLD and age range between 18 to 65 years. The exclusion criteria include history of advanced chronic liver disease, renal failure or any other active gastrointestinal disorder, Alcohol drinking, consumption of anticoagulants, weight-lowering agents, oral medications for diabetes mellitus, hepatotoxic medications and/or concurrent supplementation with vitamins or antioxidants. In all of the participants, NAFLD diagnosis was confirmed by expert gastroenterologist based on chronic elevation of liver enzymes, absence of alcohol consumption and an ultrasonography report of nonalcoholic fatty liver. Overall, 62 patients included and randomly divided into intervention or placebo groups (wheat flour), respectively. For all of them lifestyle modification advised. Then, the participants in intervention group supplemented with Turmeric (2 gr/daily) as oral capsules and the other group received placebo. The intervention period was 8 weeks and subjects were advised to consume their capsules after main meals to enhance absorption in the small intestine due to the presence of dietary fat. The medical history, demographic data, and diet habits of each patient were recorded by using a self-administered questionnaire at the beginning of the study. All of the participants had a weekly phone call to remind them about the supplements and they were also asked to report any adverse effects.

To evaluate dietary intake, including total energy, fat, protein, and carbohydrate, 24-h food recalls for three days (2 working days and a holiday) were obtained from all subjects before and after intervention. Nutrient intakes of the subjects were analyzed by using modified Nutritionist IV software (version 3.5.2, First Data Bank; Hearst Corp, San Bruno, CA). In order to height and weight of the participants, a measuring tape and digital scale were used, height was recorded with an accuracy of 0.1 cm while the participants were in standing and an upright position without shoes and weight was measured with minimal clothing and without shoes, with a precision of 0.1 kg. Body mass index (BMI) was calculated as weight in kilograms divided by height in square meters. Waist circumference (WC) and hip circumference (HC) were measured by using a non-stretchable tape, without any pressure applied to the surface of the body. These measurements were recorded with a precision of 0.1 cm. After recording of anthropometric data, blood samples obtained from all of the participants before and after the 8 weeks’ intervention period. Blood samples were kept into evacuated tubes and serum of each sample was separated after centrifugation (3000 rpm, 4 °C, 15 min) by a trained examiner. Afterwards, they were stored freezer (− 70 °C) until analysis. To avoid any effect from hormonal variation, blood samples were not collected from women during their menstrual period. Hematological factors including alanine aminotransferase (ALT), gamma-glutamyl Trans peptidase (GGT), aspartate aminotransferase (AST) and lipid profile including high-density lipoproteins (HDL), low-density lipoprotein (LDL) and very low density lipoprotein (VLDL) were determined by an automated biochemical analyzer (Hitachi-7180E, Tokyo, Japan) with a Pars Azmoon reagent kit (Tehran, Iran). The Malondialdehyde (MDA) level was determined by turbidimetric immunoassay (LDN Co., Germany). Liver Ultrasonography were performed with a 3.5/5 MHz probe at the entry and end of the study period by an expert radiologist blinded to the group allocation of the patients (General Electric LOGIQ 400 CL). The grade of hepatic steatosis, defined as the percentage of hepatocytes with fat droplets, was measured for each patient and then the degree of steatosis was categorized using the following scale: 0 (normal), 1 (mild), 2 (moderate), 3 (severe).

### Statistical analysis

All statistical analyses were performed with the Statistical Package for Social Sciences (SPSS Inc., Chicago, IL) version 18 for Windows. The normal distribution of all variables was checked with the Kolmogorov–Smirnov test. We compared the means of variables of each group with using both independent sample t tests and analysis of covariance in the adjusted models. The end values of each variable were also compared with the baseline values using paired sample t tests. Differences with P values < 0.05 were considered significant.

## Results

**Fig. 1:**
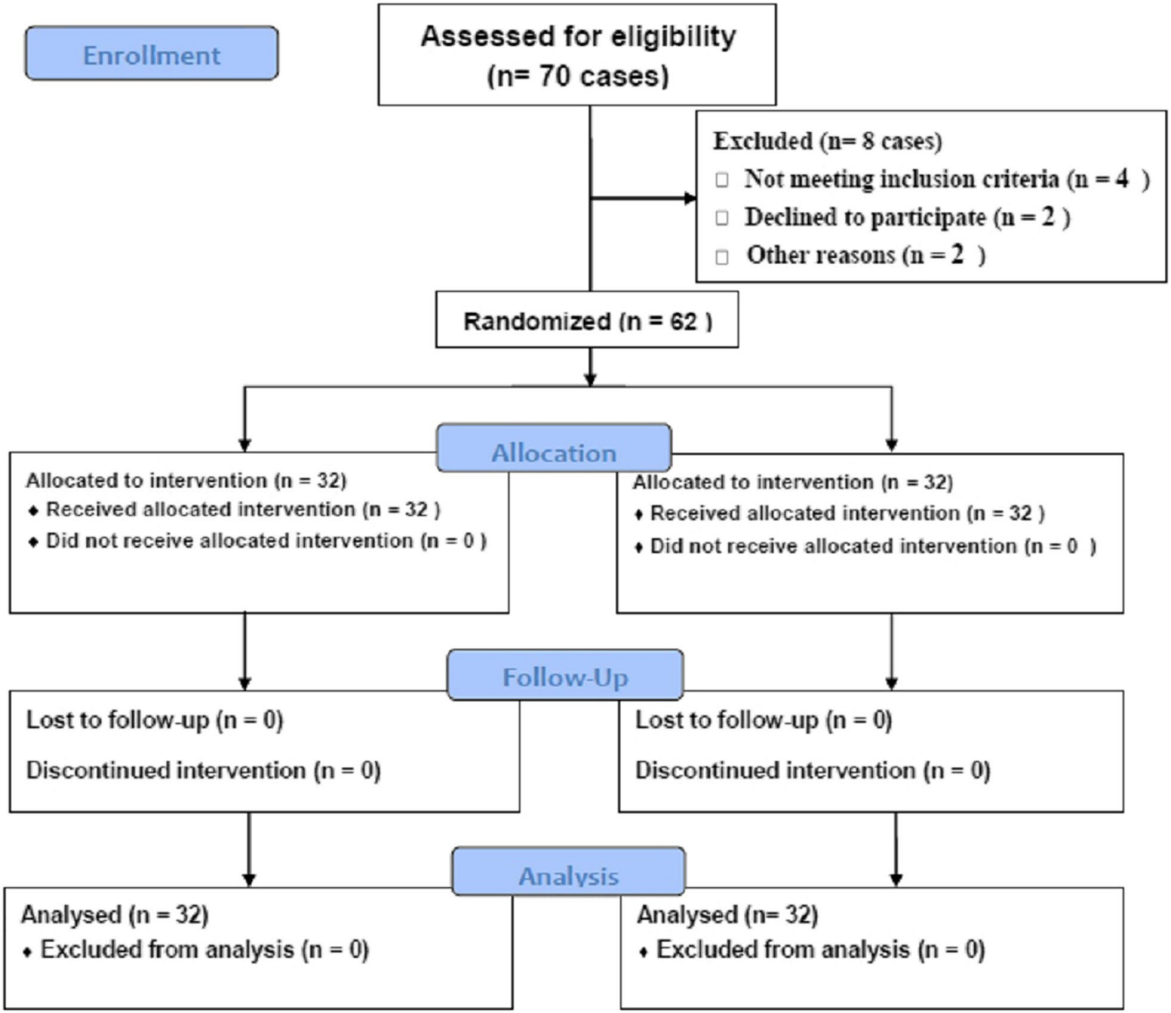
Study flow diagram

The baseline demographic, dietary intakes and anthropometric characters of two groups were similar (Table 1). After the study period, there was a significant decrease in the values such as weight, waist and WHR without any significant differences between 2 groups. Flowchart of the current research is shown in Fig. 1.

**Table 1:** Baseline characteristics of participants enrolled

Variable	Turmeric group (n = 32)	Placebo group (n = 32)	P value
Age (years)	44.12 ± 8.35	38.56 ± 10.43	0.22
Sex(M/F)	19/13	19/13	1.000^α^
Smoking	3 (9.3%)	2 (6.25%)	1.000^β^
Height (cm)	167.84 ± 11.66	167.67 ± 9.15	0.948
Body weight (kg)	82.81 ± 14.37	85.16 ± 18.47	0.572
WC (cm)	104.72 ± 10.71	105.69 ± 15.06	0.768
HC(cm)	110.55 ± 9.10	110.61 ± 10.15	0.979
WHR	0.94 ± 0.04	0.95 ± 0.06	0.722
BMI (kg/m2)	29.51 ± 4.96	30.15 ± 5.10	0.613
SBP (mmHg)	129.00 ± 16.96	124.97 ± 16.97	0.346
DBP (mmHg)	81.84 ± 8.38	81.18 ± 9.38	0.769
Energy (kcal/day)	2199.0 ± 625.2	2059.9 ± 620.2	0.375
Protein (g/day)	91.52 ± 40.87	76.13 ± 24.17	0.072
Fat (g/day)	70.38 ± 32.64	62.46 ± 29.17	0.310
Carbohydrate (g/day)	312.16 ± 11.77	305.52 ± 10.41	0.812

Values presented as mean ± SD

*M* male, *F* female, *SBP* systolic blood pressure, *DBP* diastolic blood pressure, *WC* waist circumference, *HC* hip circumference, *WHR* waist-to-hip ratio, *BMI* body mass index

^1^Analyzed by Independent t-test

^2^Values are presented as mean ± SD

^α^Analyzed by Pearson χ2

^β^Analyzed by Fisher’s exact-test

The levels of liver transaminases including ALT and AST in turmeric group were 39.56 ± 22.41 and 26.81 ± 10.54 which decreased to 30.51 ± 12.61 and 21.19 ± 5.67 respectively and in comparison with placebo group showed a significant difference (P = 0.043 and 0.044) (Table 2). After 8 weeks’ intervention, the serum levels of triglycerides (P = 0.043), LDL (P = 0.035), HDL (P = 0.049) and Malondialdehyde (MDA) (P = 0.0001) decreased in the turmeric group as compared to control group but in comparison with placebo group, these changes were nonsignificant. There were no significant changes in serum levels of total cholesterol (p = 0.196) and VLDL (P = 0.417) (Table 3). Sonographic degree of fatty liver did not reduce markedly in the turmeric group (P = 0.271) (Table 4); which could be related to short study period. However, intragroup differences in the turmeric-treated group showed a significant reduction in the percentages of NAFLD grades (p = 0.020, Table 2).

**Table 2:** Effects of Turmeric on Serum Levels of Liver Enzymes in Patients with NAFLD

Variables	Placebo, n = 32	Turmeric, n = 32	P1^a^
FBS (mg/dL)			
Before	100.70 ± 12.62	105.16 ± 12.85	0.167
After	97 ± 10.94	101.08 ± 10.51	0.134
P2^b^	0.002	0.060	
ALT (U/L)			
Before	35.42 ± 18.51	39.56 ± 22.41	0.424
After	39.50 ± 21.15	30.51 ± 12.61	0.043
P2	0.307	0.038	
AST (U/L)			
Before	27.29 ± 11.53	26.81 ± 10.54	0.861
After	25.26 ± 9.66	21.19 ± 5.67	0.044
P2	0.303	0.021	
GGT(U/L)			
Before	32.91 ± 20.26	33.81 ± 17.50	0.767
After	31.59 ± 16.70	25.62 ± 9.88	0.046
P2	0.562	0.000	
AST/ALT ratio			
Before	0.829 ± 0.23	0.744 ± 0.23	0.146
After	0.763 ± 0.05	0.773 ± 0.21	0.887
P2	0.198	0.449	

Values presented as mean ± SD

*α* analyzed by Pearson χ2, *β* analyzed by Fisher’s exact-test), *NAFLD* nonalcoholic fatty liver disease, *FBS* fasting blood sugar, *AST* aspartate aminotransferase, *ALT* alanine aminotransferase, *GGT* gamma glutamyl transpeptidase

^a,b^P values indicate differences between the intervention and placebo groups at before and after intervention (Paired T Test)

**Table 3:** Effects of turmeric on serum levels of lipid profile and malondialdehyde in patients with NAFLD

Variables	Placebo, n = 32	Turmeric, n = 32	P1
Triglycerides (mg/dL)			
Before	176.44 ± 91.96	164.34 ± 80.12	0.577
After	155.62 ± 85.35	141.78 ± 65.57	0.470
P2	0.188	0.043	
Total Cholesterol (mg/dL)			
Before	194.50 ± 34.29	195.88 ± 35.38	0.875
After	182.62 ± 29.36	186.50 ± 36.49	0.641
P2	0.143	0.196	
LDL (mg/dL)			
Before	116.52 ± 27.11	121.93 ± 28.77	0.442
After	104.60 ± 22.99	108.40 ± 26.83	0.545
P2	0.052	0.035	
HDL (mg/dL)			
Before	42.45 ± 10.17	40.28 ± 6.66	0.316
After	44.59 ± 7.17	42.34 ± 4.13	0.129
P2	0.247	0.049	
VLDL(mg/dL)			
Before	36.17 ± 14.82	30.88 ± 15.02	0.161
After	33.63 ± 16.55	32.53 ± 16.52	0.791
P3	0.394	0.417	
Cholesterol/HDL ratio			
Before	4.71 ± .0.97	5.03 ± 1.37	0.294
After	4.16 ± 0.80	4.45 ± 1.00	0.211
P2	0.012	0.021	
LDL/HDL ratio			
Before	2.81 ± 0.72	3.12 ± 0.94	0.155
After	2.38 ± 0.59	2.57 ± 0.66	0.235
P2	0.005	0.003	
MDA(mmol/L)			
Before	0.278 ± 0.14	0.237 ± 0.13	0.241
After	0.189 ± 0.13	0.167 ± 0.10	0.487
P2	0.191	0.000	

Values presented as mean ± SD

*NAFLD* nonalcoholic fatty liver disease, *LDL* low-density lipoprotein, *HDL* high-density lipoprotein, *VLDL* very low-density lipoprotein, *Unit* µM/L

^a,b^P values indicate differences between the intervention and placebo groups at before and after intervention (Paired T Test)

**Table 4:** Effects of turmeric on NAFLD degree in patients with NAFLD

NAFLD degree	Placebo n = 32	Turmeric n = 32	P1
Before intervention^α^			0.787
Normal	0	0	
Mild	23(71.9%)	24(75%)	
Moderate	8(25%)	7(21.9%)	
Severe	1(3.1%)	1(3.1%)	
After intervention			0.271
Normal	1(3.1%)	2(6.2%)	
Mild	24(75%)	26(81.2%)	
Moderate	7(21.9%)	4(12.5%)	
Severe	0	0	
P2	0.102	0.020	

*NAFLD* nonalcoholic fatty liver disease

^α^Data are expressed as n (%). P1 resulted from Mann–Whitney U test between the 2 groups and P2 resulted from Wilcoxon test within each group

## Discussion

We conducted a RCT of the efficacy of turmeric on some parameters of lipid profile, oxidative stress, liver echogenicity and liver functional test (AST, ALT, and GGT) among NAFLD patients. Overall the results of our study showed that supplementation with turmeric extracts (2000 mg/day) could reduce serum levels of ALT and AST. Elevated blood ALT and AST are conventional indicators of liver injury and usually measured in investigations on liver disease [34]. As mentioned, a combination of insulin resistance, oxidative stress, lipid peroxidation and inflammation are involved in pathogenesis of NAFLD [35–37]. Hence, any compound that controls all of these disorders could consider as a liver-protective compound. In the current research, supplementation with Turmeric significantly reduced serum levels of AST, ALT, and GGT. These findings was in agreement with two recent systematic reviews and meta-analyses that show the beneficial impact of turmeric and its active component, curcumin supplementation on reduction of serum ALT levels in subgroups with ≥ 1000 mg/day as well as serum levels of AST in studies with 8-weeks administration [24, 25]. Moreover, another meta-analysis of 4 randomized controlled trials (RCTs) indicated a considerable effect of the curcumin supplementation on lowering AST levels compared to the placebo; while, there was no significant change in ALT blood concentrations following curcumin consumption [21].

The current study also observed that turmeric supplementation for 8 weeks significantly reduced the MDA blood concentrations as a marker of lipid peroxidation, compared to baseline in patients with NAFLD. Consistent with our findings, Jakubczyk et al. recently in a meta-analysis of four RCTs demonstrated that the administration of Pure curcumin (645 mg/67 days) resulted in a significant reduction in the level of MDA as well as a increment in total antioxidant capacity [38]. Another systematic and meta-analysis reported the efficacy of purified curcuminoids supplementation in preventing the effects of oxidative stress by a considerably reduction in MDA concentrations [39]. In this case, Acar and colleagues have also addressed the ability of curcumin administration to reduce the serum MDA levels in diabetic patients [40].

The current study revealed that taking turmeric‐containing supplement for an 8-week period resulted in a significant decrease in the degree of steatosis in compare to the baseline. Given that, liver ultrasonography is a safe, inexpensive, non-invasive, and well tolerated procedure that is considered as the first-line to diagnose the severity of fatty liver diseases in the clinical and epidemiological setting [41]. In this regard, reports have illustrated considerable evidence the hepato-protective effects of turmeric to alleviate hepatic steatosis and prevent the progression of fatty liver disease in other models of hepatic dysfunction [21, 25, 41–43]. However, due to limited studies with long-term duration we should interpret our findings with caution. More studies are needed to clarify the efficacy of curcumin in different duration of intervention. Given the available data, the efficacy of curcumin on fatty liver diseases is still unclear in clinical trials, which needs to further research and consideration [20]. Through multiple pharmacological mechanisms, the liver-protecting effects of curcumin in vitro and in vivo studies has been highlighted. The anti-oxidant ability of curcumin in scavenging reactive oxygen species, reactive nitrogen species and lipid radicals is one of the most important one [44, 45]. Role of oxidative stress and inflammation in inducing hepatocyte injury and progression of NAFLD have been determined in previous studies [18, 46, 47]. Curcumin, a lipid-soluble antioxidant which is located in cell membrane reacts with lipid radicals and turns to phenoxyl radical. After that it travels to the surface of the membrane and can be neutralized by water-soluble antioxidants like vitamin C [48]. Treatment with curcumin also enhances the activities of detoxifying enzymes such as glutathione-S-transferase, glutathione peroxidase, glutathione reductase, catalase and heme-oxygenase-1 in liver as well as suppression the hepatic protein expression of oxidative stress [24, 26, 49–51]. Curcumin blocks the activation of major mediators of cellular inflammation such as NF-κB, 5-lipoxygenase (5-LOX) and cyclooxygenase-2 (COX-2); which is implicated in the activation of many genes including several pro-inflammatory and cytotoxic cytokines such as TNF-α, IL-1, IFN-γ and IL-12 [52–54]. NF-κB can also modulate the production of inducible nitric oxide synthase (iNOS), an enzyme which is involved in production of nitric oxide and hepatocyte toxicity and same as cardio-protective effects in case MI among diabetics [55, 66]. Curcumin also inhibits activation and proliferation of hepatic stellate cells which have a well-known role in progression of liver fibrosis. Decreased in liver hydroxyproline content and downregulating of collagen mRNA synthesis after curcumin treatment supports this claim [56, 57, 65]. No severe adverse events were reported in our study and even available evidence. There were only one case with stomachache and two cases with combined stomachache and nausea in Rahmani et al. trial [58].

Additionally, the results of this clinical trial demonstrated that the 2 g turmeric use have a favorable effect on serum levels of TG and LDL-c. There was also a trend toward significant increase of HDL concentrations in NAFLD patient with turmeric supplementation; but no significant difference was observed in TC and VLDL serum levels. Obesity, metabolic syndrome, diabetes and dyslipidemia are implicated in pathogenesis of NAFLD and nonalcoholic steatohepatitis (NASH) [59]. Previous studies showed beneficial effects of curcumin on insulin resistance, serum glucose, body fat and serum lipids [58, 60, 61]. Improvement in these disorders could be an effective way in management of liver disease. A study by Adab et al. revealed a significant reduction in TG and LDL-c in patients with type 2 diabetes (T2DM) after 8-weeks administration of 2100 mg turmeric powder daily; whereas, no considerable changes were seen on TC and HDL-c [39]. In contrast, a recent meta-analysis of 9 RCTs suggested curcumin supplementation effective in lowering serum TC, LDL, FBS, and waist circumference (WC) in patients with NAFLD, although not in serum TG, HDL, and body mass index (BMI) [25]. Previous systematic review and meta-analysis also represented that there was a trend to significant decrease of LDL-C, TG, FBS levels, and weight in NAFLD subjects following curcumin supplementation; however, no statistical significance change in TC and HDL- c was achieved with curcumin consumption [21]. Contrary to the results of our study, Pakfetrat et al. could not find any significant change in lipid profiles in end-stage renal disease patients who were supplemented with 1.5 g turmeric for 8 weeks [62]. In another human study, Kim et al. found that 12 weeks of fermented turmeric powder (FTP) supplementation at a dosage of 3.0 g/day no effect on serum levels of TG, total cholesterol, LDL-c, HDL-c in patients with mild to moderate elevated ALT levels [31]. Nevertheless, the sample sizes were relatively small and the obtained results were controversial. Besides, different types, dosage and preparation methods of turmeric were used among included studies, explaining that several functional compounds in turmeric may involve in its beneficial effects on liver.

Based on the RCTs, there was several plausible mechanisms in the estimates supporting favorable effect of curcumin for lowering TG and LDL-c levels and also prevention hepatic lipid accumulation [25]. A possible mechanism may be related to metabolites of absorbed curcuminoids; which can play as ligands to activate the expression of the peroxisome proliferator-activated receptors (PPARs) gene that implicated in intra- and extracellular lipid metabolism [63]. Considering that, PPARs play a central role in a signaling system that control lipid homeostasis. It seems that lowering serum TG and LDL-c levels are also related probably owing to a curcumin-induced increase in the expression of several enzymes involving fatty acid metabolism such as cholesterol 7a-hydroxylase, hemeoxygenase-1, and low density lipoprotein receptors and a similar decrease in the expression of HMG-CoA reductase [21]. Moreover, curcumin could lead to lowering plasma LDL-c via reduction of cholesteryl ester transfers protein (CETP) from HDL and/or enhanced clearance of plasma LDL-c. Curcumin-induced reduction of CE transfer between lipoproteins due to CETP inhibition results in decreasing LDL-c with simultaneous increasing HDL-c concentration [64].

Several limitations existed in our study. Firstly, due to ethical considerations, we could not use liver biopsy which is a more accurate diagnostic tool. Secondly, follow-up duration was not long enough to consider the effects of turmeric on the hepatic system. Thirdly, we could not exactly evaluate the loyalty of the participants to the treatments but we controlled this problem, by repeated follow-up visits and by counting the capsules.

## Conclusion

In conclusion the results of our study showed that supplementation with turmeric extracts reduce elevated serum levels of ALT and AST among patients with NAFLD. Decreasing of these two enzymes could indicate improvement in liver function. Therefore, it could be considered as a good adjuvant therapeutic supplement with hypo lipidemic and antioxidant properties for this disease. However, more well-designed randomized clinical trials are needed to investigate other indicators of NAFLD. Furthermore, the beneficial role of curcumin in other liver diseases remained unclear due to the lack of trials on these populations.

## Data Availability

The datasets analyzed during the current study are available from the corresponding author on reasonable request.

## Abbreviations

NAFLD: Nonalcoholic fatty liver disease
MDA: Malondialdehyde
AST: Aspartate transaminase
ALT: Alanine transaminase
GGT: Gamma-glutamyl transferase
LDL: Low-density lipoproteins
HDL: High-density lipoproteins
VLDL: Very-low-density lipoprotein
NASH: Nonalcoholic steatohepatitis
BMI: Body mass index
HC: Hip circumference
WC: Waist circumference
iNOS: Inducible nitric oxide synthase
IL: Interleukin
PPARs: Peroxisome proliferator-activated receptors
HMG-CoA: 3-Hydroxy-3-methyl-glutaryl-coenzyme A
CETP: Cholesteryl ester transfers protein
